# Factors Affecting Adherence to Social Distancing among Adults Aged 19–44 Years: Insights from a Nationwide Survey during COVID-19 Pandemic

**DOI:** 10.3390/medicina60050827

**Published:** 2024-05-17

**Authors:** Eun Jung Kim, Mikyong Byun

**Affiliations:** 1 Department of Nursing, Seoil College, Seoul 02192, Republic of Korea; ejissy@g.seoil.ac.kr; 2 Department of Nursing, Deajeon University, 62, Daehak-ro, Dong-gu, Daejeon 300716, Republic of Korea

**Keywords:** COVID-19, social distancing, compliance

## Abstract

*Background and Objectives*: Before COVID-19 vaccinations became available, adhering to non-pharmaceutical interventions (NPIs), like social distancing (SD), wearing masks, and hand hygiene, were crucial to mitigating viral spread. Many studies reported that younger individuals were more reluctant to follow these measures compared with older ones. We hypothesized that it would be worthwhile to find factors that influenced SD compliance among young people during the pre-vaccination phase of a pandemic. *Materials and Methods*: We analyzed data of adults aged 19–44 from the 2020 South Korean Community Health Survey and compared socio-demographic, health-related behavioral, and psychological factors between compliant and non-compliant cohorts. *Results*: A total of 59,943 participants were enrolled and we found that older age groups (30–39 and 40–44) and safety concerns (such as viral infection, virus-related death, economic damage, and transmitting virus to vulnerable people) were significantly associated with adherence to SD. Conversely, participants who were not living with a spouse, were unable to stay at home despite symptoms, smoked, drank, and had a negative attitude toward government policy statistically correlated with non-compliance. *Conclusions*: In times when NPIs were the primary defense against the pandemic, it is essential to identify factors that positively or negatively affect individual compliance with them, especially among young people. Using a large-scale, well-designed national survey, we could gain insights into the early recognition of risk factors for non-compliance and appropriate follow-up interventions (i.e., education campaigns, clear communication of public guidelines, and implementation of guidelines), which will help people to avoid suffering from other waves of future infectious diseases.

## 1. Introduction

The coronavirus disease 2019 (COVID-19) produced the 2020–2023 pandemic that infected approximately 775 million people, with a mortality rate of 0.009%, equaling 7 million people [1]. This deadly virus, first identified during the Chinese outbreak in December 2019, rapidly spread worldwide in early 2020. The World Health Organization (WHO) declared it a public health emergency of international concern (PHEIC) in January 2020. By March 2020, the WHO acknowledged this outbreak as a pandemic. This PHEIC designation persisted until May 2023 [2]. COVID-19, caused by SARS-CoV-2, manifests with a spectrum of symptoms, ranging from asymptomatic cases to severe illness and death. The most common symptoms include fever, sore throat, cough, and fatigue [3]. The virus mainly spreads through contact with respiratory droplets from someone infected [4]. Transmission through contaminated surfaces was proposed, but there is also controversy over this route of infection [5,6]. As the pandemic unfolded, governments and health authorities implemented various non-pharmaceutical interventions (NPIs) to minimize the viral spread [7]. These measures included hand hygiene, use of personal protective equipment, wearing masks, maintaining a distance of at least two meters from one another, and limiting travel and social gatherings [8,9,10]. As knowledge about viral transmission improved and vaccines became available, wiping down surfaces, wearing gloves and gowns, and handwashing were less emphasized [11].

Over time, SARS-CoV-2 mutations have given rise to numerous variant strains with varying degrees of infectivity and virulence [12]. In response, vaccination campaigns were launched worldwide, beginning in December 2020, aiming to curb the number of COVID-19 cases and deaths from viral infection [13]. In Korea, the coronavirus vaccine was first introduced in February 2021, and it took a long time before it was widely administered to the public [14]. Thus, NPIs, such as actively practicing social distancing, as well as personal protection, were the only available strategies to lower infection mortality rates in the early stages of the pandemic when there were no available pharmaceuticals [15,16]. The definition of social distancing varies depending on the situation, but the universally accepted explanation is the one from the Centers for Disease Control and Prevention (CDC) that suggests ‘limiting face-to-face contact with others is the best way to reduce the spread of COVID-19’ [17]. The implementation of social distancing measures ranged from government-driven lockdown policies, like prohibiting massive social gatherings, to community-driven crowd management in indoor spaces. To some extent, these social distancing methods have been effective in controlling the spread of the virus [18,19]. However, individuals reported increased fatigue, depression, anxiety, and decreased quality of sleep and physical activity when they abide by social distancing [20,21]. Despite the initial distrust of its effectiveness and side effects, COVID-19 vaccines gradually proved effective regarding COVID-19 [22]. As a result, public adherence to social distancing gradually decreased over time [23]. Factors related to compliance with social distancing policies were sex, age, marital status, education level, income, occupation, smoking, drinking, trust in government, and individual concerns about COVID-19 [24,25,26,27,28,29,30]. Old-aged adults demonstrated greater compliance, but young-aged ones, especially adults aged 19–44 years, exhibited lower levels of adherence [31,32]. They perceived their chances of getting infected and suffering from severe illness to be low [20,33].

As infectious diseases similar to COVID-19 may spread globally again, it is very important to learn lessons from the recent pandemic. In times when vaccinations are not available and NPIs are the only means of defense against the pandemic, it is important to identify factors that positively or negatively affect an individual’s compliance with social distancing, especially in young age groups. When we pay attention to individuals who are reluctant to socially distance, they are readily found among various types of surveys. In this study, we hypothesized that we could gain in-depth insights into social distancing compliance and related factors by utilizing a large-scale, well-designed national survey. First, we compared socio-demographic, health-related behavioral, and psychological factors between compliant and non-compliant cohorts using public data. We then aimed to find statistically significant variables associated with adherence to social distancing through regression analysis.

## 2. Materials and Methods

### 2.1. Data Source

Our data were derived from the 2020 Korea Community Health Survey, which conducted additional investigations on COVID-19 [34]. This survey took place through computer-assisted in-person interviews from August to October 2020. It involved 229,269 community-dwelling adults aged 19 or older.

### 2.2. Participant Selection and Study Design

From the original data, we retrieved 67,701 adults aged 19 to 44 years. After excluding 7758 missing or incomplete responses, a total of 59,943 individuals were eligible for this study. We assessed the predictive factors for social distancing using three (socio-demographic, health-related behavioral, and psychological factors) categories based on a descriptive and cross-sectional design. Afterward, we determined essential variables in terms of compliance with social distancing using multiple logistic regression.

### 2.3. Measurements

#### 2.3.1. Social Distancing

Social distancing was defined as the act of refraining from going out and avoiding gatherings or events in the week prior to the survey being conducted [34]. Compliance with social distancing was determined using the following question, ‘Have you practiced social distancing in the past week? In other words, did you refrain from going out and avoid gatherings and events?’ Participants could answer ‘Yes’ or ‘No’, which was classified as compliance and non-compliance, respectively.

#### 2.3.2. Socio-Demographic and Health-Related Behavioral Factors

Participants’ socio-demographic characteristics included age, marital status, education level, monthly household income, occupational status, and the ability to stay at home despite symptoms such as a high fever and cough. Health-related behaviors involved current smoking (yes or no) and alcohol consumption.

#### 2.3.3. Psychological Factors

Personal perceptions of COVID-19 included variables such as concerns about infection, death, criticism from others, economic damage, transmitting the virus to vulnerable people, and individual thoughts on the appropriateness of the government’s response to the pandemic. One question was used to detect the presence of a particular factor, and the results of each response were classified into ‘No’, ‘Neutral’, and ‘Yes’. Personal thought on the appropriateness of the government’s response was divided into ‘Appropriate’, ‘Neutral’, ‘Inappropriate’, and ‘Unknown or refused to respond’.

### 2.4. Data Analysis

We performed descriptive statistics using the χ^2^ or t-test to compare differences in the social distancing compliance status in view of the socio-demographic, health-related behavioral, and psychological aspects (a *p*-value less than 0.05 was statistically significant). Initially, each independent factor was analyzed for univariate logistic regression, and then, statistically significant factors were selected for carrying out multivariate logistic regression. Odds ratios (ORs) and the corresponding 95% confidence intervals (CIs) are also presented. The level of statistical significance was set at a *p*-value less than 0.05. The data were analyzed using the IBM SPSS version 28.0 software package (IBM, Armonk, NY, USA).

## 3. Results

### 3.1. Prevalence of Social Distancing in Adults Aged 19–44 Years

A total of 59,943 participants were enrolled and nearly all of them (96.3%, n = 57,726) were adherent to social distancing rules.

### 3.2. Differences in Socio-Economic and Health-Related Behavioral Factors between Social Distancing Compliant and Non-COMPLIANT Groups

More than half (51.8%, n = 31,053) were female. Adults aged 19–29, 30–39, and 40–44 years accounted for 39.1%, 37.2%, and 23.7%, respectively. People who were living with a spouse accounted for 44.5%. Most participants had a college degree or higher (76.7%) and were paid workers (57.2%). The vast majority were able to stay at home when they had a fever or cough (92.1%). In terms of health-related behaviors, most of them did not smoke (80.2%) and 21.9% did not drink at all (Table 1). In detail, there were more women (52.1%) in the social distancing (SD) group and there were more men in the non-social distancing (NSD) group (54.5%) (χ^2^ = 37.03, *p* < 0.001). In the SD group, 40–44 years old participants recorded the lowest number of participants (24.1%), while those aged 19–29 years old recorded the highest number (56.8%) in the NSD group (χ^2^ = 322.02, *p* < 0.001). The majority of the participants did not live with their spouses (SD = 55.5%, NSD = 68.7%) (χ^2^ = 163.93, *p* < 0.001), and earned a relatively high household income (SD = 42.3%, NSD = 42.9%) (χ^2^ = 9.01, *p* = 0.011). If they had a high fever or cough, most of the participants could stay and rest at home (SD = 92.2%, NSD = 88.6%) (χ^2^ = 37.59, *p* < 0.001). The majority of the participants were not smokers (SD = 80.5%, NSD = 72.0%) (χ^2^ = 95.29, *p* < 0.001) and were non-drinkers (SD = 52.4%, NSD = 37.4%) (χ^2^ = 228.97, *p* < 0.001).

In short, concerning socio-demographic and health-related behavioral variables, seven items (sex, age, living with spouse, monthly income, ability to stay at home despite symptoms, smoking, and drinking) showed statistical significance between compliant and non-compliant groups.

### 3.3. Differences in Psychological Factors between Social Distancing Compliant and Non-Compliant Groups

The differences in social distancing adherence according to psychological variables are summarized in Table 2. The majority of participants (66.2%) were concerned about getting infected, and 33.0% were concerned about dying. More than half of all participants were concerned about being blamed by others (70.7%), economic harm (72.9%), and infecting vulnerable populations (78.9%). The majority (69.2%) thought the government’s response to the pandemic was appropriate. Most participants were more concerned about infection than death related to the virus in the SD (66.6% and 33.5%, respectively) and NSD (54.9% and 20.8%, respectively) groups (χ^2^ = 205.95, *p* < 0.001; χ^2^ = 229.64, *p* < 0.001, respectively). Most of them were concerned about receiving criticism from others in the group that practiced SD (70.9%) and in the group that did not (65.0%) (χ^2^ = 66.93, *p* < 0.001). Most participants answered ‘Yes’ to having concerns about economic damage in the SD (73.2%) and NSD (64.3%) groups (χ^2^ = 121.78, *p* < 0.001). Participants were also concerned about infecting vulnerable individuals in the SD (79.3%) and NSD (69.2%) groups (χ^2^ = 179.03, *p* < 0.001). Participants who felt the government’s response to the COVID-19 pandemic was appropriate accounted for 69.5% in the group that practiced SD and 61.6% in the group that did not (χ^2^ = 84.45, *p* < 0.001). Most participants were more concerned about infection than death related to the virus in the SD (66.6% and 33.5%, respectively) and NSD (54.9% and 20.8%, respectively) groups (χ^2^ = 205.95, *p* < 0.001; χ^2^ = 229.64, *p* < 0.001, respectively). A lot of them were concerned about receiving criticism from others in the group that practiced SD (70.9%) and in the group that did not (65.0%) (χ^2^ = 66.93, *p* < 0.001). Most participants answered ‘Yes’ to having concerns about economic damage in the SD (73.2%) and NSD (64.3%) groups (χ^2^ = 121.78, *p* < 0.001). Participants were also concerned about infecting vulnerable individuals in the SD (79.3%) and NSD (69.2%) groups (χ^2^ = 179.03, *p* < 0.001). Participants who felt the government’s response to the COVID-19 pandemic was appropriate, accounted for 69.5% in the group that practiced SD and 61.6% in the group that did not (χ^2^ = 84.45, *p* < 0.001).

### 3.4. Multivariable Logistic Regression Analysis of Socio-Demographic, Health-Related Behavioral, and Psychological Factors Affecting Adherence to Social Distancing

The SD compliance rate for adult participants aged 30–39 years (odds ratio [OR] = 1.77, *p* < 0.001) and 40–44 years old (OR = 2.56, *p* < 0.001) were higher than that of adults aged 19–29 years. It was lower for participants who were not living with their spouses (OR = 0.87, *p* = 0.025), were unable to stay at home when showing symptoms (OR = 0.64, *p* < 0.001), and were current smokers (OR = 0.77, *p* < 0.001) compared with those who were none of the above. Compliance was higher among participants who drank less than once a week (OR = 0.62, *p* < 0.001) and those who drank twice or thrice a week (OR = 0.44, *p* < 0.001) than those who drank more than four times weekly (OR = 0.39, *p* < 0.001). The compliance rate of social distancing was higher among young adults who answered ‘Neutral’ (‘N’ hereafter) and ‘Yes’ (‘Y’ hereafter) to having concerns about infection (N: OR = 1.17, *p* = 0.031; Y: OR = 1.26, *p* = 0.002), death (N: OR = 1.27, *p* < 0.001; Y: OR = 1.71, *p* < 0.001), economic damage (N: OR = 1.20, *p* = 0.023; Y: OR = 1.35 *p* < 0.001), and infecting vulnerable people (N: OR = 1.24, *p* = 0.035; Y: OR = 1.35, *p* < 0.001). The compliance rate of those who answered ‘Neutral’ (OR = 0.87, *p* = 0.006) and ‘Inappropriate’ (OR = 0.70, *p* < 0.001) regarding the appropriateness of the government’s response was lower than that of those who answered ‘Yes’ (Table 3).

In summary, there were statistically significant differences between the two groups in view of psychological factors, such as fear of infection, virus-related deaths, blame from others, economic harm, the likelihood of infecting vulnerable populations, and the adequacy of the government’s response to the pandemic and its aftermath.

## 4. Discussion

As mentioned before, COVID-19 vaccines were first introduced to Koreans in February 2021, and it took a while before they were widely administered to the general population [14]. Until the introduction of vaccinations, NPIs, such as social distancing and personal protective measures, such as wearing facial masks, were recommended or even forced directly or indirectly onto individuals to mitigate viral spread [7]. To promote compliance with social distancing measures, emphasis was placed on educational campaigns to promote public awareness, clear communication of public health guidelines, and the implementation of policies and regulations [35,36]. Social distancing as a part of NPIs was also bound to have its side effects, where some researchers suggested the use of the term ‘distant socializing’ instead of ‘social distancing’ because the latter may imply that individuals should stop socializing [37]. We have to keep in mind that these interventions inevitably restricted individual freedom to some extent, where many studies reported those who were reluctant to follow official regulations were more frequently found in younger populations than in older ones [32,38,39]. Koreans also experienced the same phenomenon and there were well-designed studies on the topic [40,41,42], but some of them were based on relatively small cohorts or were conducted after the introduction of vaccines. Using a large-scale population-based national survey related to social distancing [34], we postulated that it would be worthwhile to identify factors that might potentially influence younger people’s compliance with social restrictions in the early pandemic without available vaccines. In this study, we revealed that advanced age (30–39 and 40–44), and personal safety concerns (i.e., viral infection, virus-related death, economic damage, and transmitting the virus to vulnerable people) were significantly associated with one’s adherence to social distancing. On the other hand, individuals who did not live with a spouse, were unable to stay at home despite symptoms, smoked, drank, and had a negative attitude toward government policy statistically correlated with non-compliance with social distancing.

In view of the demographic factors, many studies uniformly suggested that younger age was one of the key factors that interfered with adherence to social distancing [24,25,26,27,28,29,30]. However, there existed no clear standards or explanations for what age group could be called ‘young’ among studies. One study designated only 20–25 years old as a young age [43], the other studies classified 18–34 or even 18–44 years old as a young age [31,32]. Since our secondary data analysis of this national survey intended to focus on the compliance comparisons between young and old people, we defined our young age group as 19- to 44-year-old adults. We also found that the compliance rate was high in participants living with their spouses and those whose responses were neutral or affirmative to concerns regarding infecting vulnerable people in the family. This might be related to the fact that marital status and the presence of children may influence one’s practicing social distancing [44]. When there are more family members in a household, the risk of infection increases, especially for children or older adults who are more vulnerable to viral transmission. Participants in their 30s and 40s who already had children or old adults in their homes more strictly followed social distancing policies to protect their family members [29,31]. Even among unmarried young adults, the rate of adherence increased when they lived with fragile family members [33]. These indicators, such as marital status, whether there were children or elderly people at home, and taking care of parents, helped to confirm the person’s family-consciousness in maintaining social distancing. However, demographic items of the national survey only contained ‘age’ and ‘living with spouse status’ [34]. Since we were not able to look at these important indicators in detail, caution may be needed when interpreting our research results as given.

Because young people participate more actively in social and economic life than older people, it is also important to review socio-economic factors that affect their adherence to social distancing between compliant and non-compliant cohorts. In our study, being able to rest at home when experiencing symptoms was statistically significantly related to compliance with social distancing, but income level was not. According to our literature review, studies pointed out that the degree of social distancing was strongly elevated in those with higher incomes who were less likely to work outside the home during the pandemic [45,46]. Researchers reported that national social distancing guidelines needed to be established and financial support might be necessary when job stability was diminished for groups who were heavily affected by the pandemic, such as non-regular workers [47].

Similar to other research findings, compliance with social distancing was significantly lower in participants whose health-related behaviors were ‘currently smoking’ or ‘drinking more than once a week’. Smokers usually tend to go outside and remove their masks to smoke, making it difficult for them to comply with social distancing [29]. Fendrich et al. commented that if one’s frequency of drinking increased, subsequent social distancing compliance and personal hygiene rate decreased [47]. Similar to our finding, researchers reported that people who drink alcohol more than once a week may not adhere to social distancing [48].

In terms of psychologic variables, we found that safety concerns (i.e., fear of infection, virus-related death, economic loss, and possible viral transmission onto helpless people) were significantly associated with social distancing adherence. As a voluntary and preventive health-related behavior, the theory of planned behavior may be useful for predicting social distancing compliance [49]. According to this theory, concerns about infection or death increase when individuals perceive higher susceptibility to the virus, leading to an increase in social distancing compliance [28,43,50]. On the other hand, participants who expressed negative attitudes to official policy statistically correlated with non-compliance. Similar results could be found in previous studies [25,29,31,33], which highlighted how trust in the government and the effective delivery of information via social media platforms had a positive effect on the implementation of preventive interventions against infectious diseases.

There were four major limitations in our study. First, there was a risk of recall bias since our data source was from the interview. Second, there were only two demographic variables (age and living with a spouse) in the original datasheet because this national survey was intended to investigate community-dwelling adults’ general health status. In other words, other demographic determinants of compliance with social distancing (i.e., one’s marital status, presence of children, or elderly people at home) [29,31,33,44] need to be considered in future studies. Third, only one question was used to evaluate compliance with social distancing, which might restrict the scope of analyzing the effects of various methods of social distancing. Finally, our major disadvantage originated from using secondary data as a backbone. It may provide unclear answers to the researcher’s research-related questions or does not contain additional information that researchers would like to investigate. Thus, special attention should be given when interpreting our secondary data analysis.

Despite these shortcomings, our data can be utilized to learn lessons on NPIs against the spread of infectious diseases in the early stages when pharmaceutical means are unavailable. Our findings necessitate further validation by well-designed studies with a larger cohort.

## 5. Conclusions

In times when non-pharmaceutical interventions are the only defense against a pandemic, it will be essential to find clues that positively or negatively influence individual compliance with preventive measures, especially among younger individuals. Using a large-scale, well-designed national survey, we could gain insights into the fact that participants who were in the youngest age group (19 to 29 years old), without safety concerns, not living with a spouse, unable to stay at home despite symptoms, currently smoking and drinking, and having a negative attitude toward government policy statistically correlated with non-compliance with social distancing. In a future pandemic, early recognition of risk factors for non-compliance and appropriate follow-up interventions (i.e., education campaigns, clear communication of public guidelines, and implementation of guidelines) will help people to avoid suffering from another wave of infectious diseases.

## Figures and Tables

**Table 1:** Differences in social distancing compliance according to participants’ sociodemographic characteristics and health-related behaviors (n = 59,943).

		Total (n = 59,943)	Social Distancing		χ^2^	*p*
			Yes (n = 57,726)	No (n = 2217)		
		n (%)	n (%)	n (%)		
Sex	Female	31,053 (51.8)	30,045 (96.8)	1008 (3.2)	37.03	<0.001
	Male	28,890 (48.2)	27,681 (95.8)	1209 (4.2)		
Age (years)	19–29	23,457 (39.1)	22,197 (94.6)	1260 (5.4)	322.02	<0.001
	30–39	22,306 (37.2)	21,644 (97.0)	662 (3.0)		
	40–44	14,180 (23.7)	13,885 (97.9)	295 (2.1)		
Living with spouse	Yes	26,687 (44.5)	25,994 (97.4)	693 (2.6)	163.93	<0.001
	No	33,256 (55.5)	31,731 (95.4)	1524 (4.6)		
Education	College and above	46,034 (76.8)	44,334 (96.3)	1700 (3.7)	0.13	0.936
	High school	13,247 (22.1)	12,753 (96.3)	494 (3.7)		
	Middle school	662 (1.1)	639 (96.5)	23 (3.5)		
Monthly income	High	14,878 (24.8)	24,443 (93.6)	952 (6.4)	9.01	0.011
	Medium	19,670 (32.8)	19,001 (96.6)	669 (3.4)		
	Low	25,395 (42.4)	14,282 (97.7)	596 (2.3)		
Occupational status	Paid worker	34,269 (57.2)	32,964 (96.2)	1305 (3.8)	3.39	0.335
	Employer or self-employed	5669 (9.5)	5478 (96.7)	191 (3.3)		
	Unpaid worker	875 (1.5)	844 (96.5)	31 (3.5)		
	Unemployed	19,130 (31.9)	18,440 (96.4)	690 (3.6)		
Able to stay at home despite symptoms	Yes	55,175 (92.1)	53,211 (96.4)	1964 (3.6)	37.59	<0.001
	No	4768 (8.0)	4515 (94.7)	253 (5.3)		
Currently smoking	No	48,044 (80.2)	46,447 (96.7)	1597 (3.3)	95.29	<0.001
	Yes	11,899 (19.9)	11,279 (94.8)	620 (5.2)		
Drinking	Never	13,128 (21.9)	12,811 (97.6)	317 (2.4)	228.97	<0.001
	≤Once a month	17,951 (30.0)	17,433 (97.1)	518 (2.9)		
	≤Once a week	16,867 (28.1)	16,155 (95.8)	712 (4.2)		
	2–3 times a week	9720 (16.2)	9189 (94.5)	531 (5.5)		
	≥4 times a week	2277 (3.8)	2138 (93.9)	139 (6.1)		

**Table 2:** Differences in social distancing compliance according to participants’ perceptions of COVID-19 (n = 59,943).

		Total (n = 59,943)	Social Distancing		χ^2^	*p*
			Yes (n = 57,726)	No (n = 2217)		
		n (%)	n (%)	n (%)		
Concerns about being infected with the virus	No	5620 (9.4)	5238 (93.2)	382 (6.8)	205.95	<0.001
	Neutral	14,666 (24.5)	14,049 (95.8)	617 (4.2)		
	Yes	39,657 (66.2)	38,439 (96.9)	1218 (3.1)		
Concerns about death related to the virus	No	23,496 (39.2)	22,307 (94.9)	1189 (5.1)	229.64	<0.001
	Neutral	16,676 (27.8)	16,110 (96.6)	566 (3.4)		
	Yes	19,771 (33.0)	19,309 (97.7)	462 (2.3)		
Concerns about receiving criticism from others	No	7809 (13.0)	7395 (94.7)	414 (5.3)	66.93	<0.001
	Neutral	9784 (16.3)	9421 (96.3)	363 (3.7)		
	Yes	42,350 (70.7)	40,910 (96.6)	1440 (3.4)		
Concerns about economic damage	No	6985 (11.7)	6574 (94.1)	411 (5.9)	121.78	<0.001
	Neutral	9253 (15.4)	8872 (95.9)	381 (4.1)		
	Yes	43,705 (72.9)	42,280 (96.7)	1425 (3.3)		
Concerns about infecting vulnerable people	No	3050 (5.1)	2824 (92.6)	226 (7.4)	179.03	<0.001
	Neutral	5590 (9.3)	5342 (95.6)	248 (4.4)		
	Yes	47,301 (78.9)	45,766 (96.8)	1535 (3.2)		
	No vulnerable family members	4002 (6.7)	3794 (94.8)	208 (5.2)		
Appropriateness of government’s response to COVID-19	Appropriate	41,501 (69.2)	40,136 (96.7)	1365 (3.3)	84.45	<0.001
	Neutral	13,836 (23.1)	13,247 (95.7)	589 (4.3)		
	Inappropriate	4442 (7.4)	4190 (94.3)	252 (5.7)		
	Unknown or no answer	164 (0.3)	153 (93.3)	11 (6.7)		

**Table 3:** Multivariable logistic regression analysis of factors associated with adherence to social distancing.

		Exp(B)	95% Confidential Interval		*p*
			Lower	Upper	
Sex	Female	1			
	Male	1.08	0.98	1.19	0.137
Age	19–29	1			
	30–39	1.77	1.58	1.98	<0.001
	40–44	2.56	2.20	2.98	<0.001
Living with their spouse	Yes	1			
	No	0.87	0.78	0.98	0.025
Monthly income	High	1			
	Medium	1.02	0.92	1.14	0.647
	Low	0.97	0.87	1.08	0.530
Ability to rest at home in case of high fever and cough	Yes	1			
	No	0.64	0.56	0.73	<0.001
Currently smoking	No	1			
	Yes	0.77	0.69	0.86	<0.001
Drinking	Never	1			
	≤Once/month	0.93	0.80	1.07	0.283
	≤Once/week	0.62	0.54	0.72	<0.001
	2–3 times/week	0.44	0.38	0.51	<0.001
	≥4 times/week	0.39	0.31	0.48	<0.001
Concerns about being infected with the virus	No	1			
	Neutral	1.17	1.02	1.36	0.031
	Yes	1.26	1.09	1.46	0.002
Concerns about death related to the virus	No	1			
	Neutral	1.27	1.14	1.42	<0.001
	Yes	1.71	1.51	1.94	<0.001
Concerns about receiving criticism from others	No	1			
	Neutral	1.13	0.96	1.32	0.140
	Yes	0.97	0.85	1.10	0.593
Concerns about economic damage	No	1			
	Neutral	1.20	1.03	1.40	0.023
	Yes	1.35	1.19	1.53	<0.001
Concerns about infecting vulnerable people	No	1			
	Neutral	1.24	1.02	1.52	0.035
	Yes	1.35	1.15	1.60	<0.001
	No vulnerable members in the family	1.13	0.92	1.39	0.257
Appropriateness of government’s response to COVID-19	Appropriate	1			
	Neutral	0.87	0.79	0.96	0.006
	Inappropriate	0.70	0.60	0.80	<0.001
	Unknown or no answer	0.65	0.35	1.21	0.176

## Data Availability

Our data are readily available upon reasonable request.
